# Selective Divalent Cobalt Ions Detection Using Ag_2_O_3_-ZnO Nanocones by ICP-OES Method for Environmental Remediation

**DOI:** 10.1371/journal.pone.0114084

**Published:** 2014-12-02

**Authors:** Mohammed M. Rahman, Sher Bahadar Khan, Hadi M. Marwani, Abdullah M. Asiri

**Affiliations:** 1 Center of Excellence for Advanced Materials Research (CEAMR), King Abdulaziz University, Jeddah, Saudi Arabia; 2 Department of Chemistry, Faculty of Science, King Abdulaziz University, Jeddah, Saudi Arabia; Dowling College, United States of America

## Abstract

Here, we have synthesized Ag_2_O_3_-ZnO nanocones (NCs) by a wet-chemical route using reducing agents at low temperature. The structural, optical and morphological properties of Ag_2_O_3_-ZnO NCs were investigated by several conventional techniques such as powder XRD, XPS, FESEM, XEDS, FTIR and UV/vis. spectroscopy. The analytical parameters of prepared NCs were also calculated for a selective detection of divalent cobalt [Co(II)] prior to its determination by inductively coupled plasma-optical emission spectrometry (ICP-OES). The selectivity of NCs toward various metal ions, including Cd(II), Co(II), Cr(III), Cu(II), Fe(III), Ni(II), and Zn(II) was studied. Results of the selectivity study demonstrated that Ag_2_O_3_-ZnO NC phase was the most selective towards Co(II) ion. The uptake capacity for Co(II) ion was experimentally calculated to be ∼76.69 mgg^−1^. Moreover, adsorption isotherm data provided that the adsorption process was mainly monolayer on homogeneous adsorbent surfaces of Ag_2_O_3_-ZnO NCs. Kinetic study revealed that the adsorption of Co(II) on Ag_2_O_3_-ZnO NCs phase followed the pseudo-second-order kinetic model. In addition, thermodynamic results provided that the adsorption mechanism of Co(II) ions on Ag_2_O_3_-ZnO NCs was a spontaneous process and thermodynamically favorable. Finally, the proposed method was validated by applying it to real environmental water samples with reasonable results.

## Introduction

Semiconductor nanomaterials are very attractive solid supporting materials due to their lower dimension and large active surface area to volume ratio as compared to the traditional materials in nano-meter scale. Semiconductor metal oxides have employed a huge deal due to their exceptional properties such as large-active surface area, high-stability, quantum confinement consequence, and high porosity as well as permeability (meso-porous nature), which is extensively dependent on their morphology and crystallinity [1]–[3]. Codoped nanomaterials have concerned regarding the potential research achievement for its exceptional and outstanding properties as well as versatile applications [4]. Recently, an extensive development has been made on the research leading of metal-oxides (semiconductor oxides) actuated by both fundamental sciences and advanced technologies [5]. The host semiconductor nanomaterials exhibit promising uses as field-effect transistors [6], UV-photo-detectors [7], bio-sensors [8]–[11], field-emission electron sources [12], doped nanomaterials [13], nano-scale power generators [14], and many other functional devices [15]. The codoped nanostructure is generally prepared by a competent wet-chemical technique to regulate the energy-level metal oxide surface states, which can further progress by changing of dopant concentration in semiconductor materials. Silver oxide materials have attracted considerable attention because of their potential dopants applications in fabricating nano-scale electronics, opto-electronics, bio-material sensors, biological devices, electron-field emission sources for emission displays, and the surface enhanced Raman properties [16]–[18]. It exhibits wide-group of derivatives that attracted significant recognition, mainly due to the widespread uses of oxides. In nanotechnology, doped materials have been playing a key-role in the development of very accurate, highly-sensitive, and reliable detectors. The nanomaterials capable of nano-level imaging and controlling of nano-material, bio-chemical, pathological samples have attained the attention of scientist, particularly for control monitoring due to their increasing needs in healthcare and environmental monitoring [19], [20]. Semiconductor nanostructure materials are being comprehensively investigated due to their unique surface properties presented by large surface areas, which can make them ideal photo-catalysts, sensor elements, and solid phase adsorbents. Lately, development of solid-adsorbent based on semiconductor doped metal oxides conducting polymers and nano-composites is major study for determination and recognition of various toxic metallic constituents in the environments [21], [22].

In addition, the development of simple, rapid and efficient methods have become of interest for monitoring metal ions in the environment. Several analytical methods have been applied to analyze metal ions in aqueous solutions, such as atomic absorption spectrometry [23], inductively coupled plasma-optical emission spectrometry (ICP-OES) [24], anodic stripping voltammetry [25], and ion chromatography [26]. However, analytical method is directly measured metal ions, in particular at ultra-trace concentration, in aqueous systems due to the lack of sensitivity and selectivity of the methods. Therefore, an efficient separation procedure is usually required prior to the determination of noble metals for sensitive, accurate and interference-free determination of noble metals [27]. Several analytical methods can be used for separation of analytes, including liquid–liquid extraction [28], ion exchange [29], co-precipitation [30], cloud-point extraction [31], and solid phase extraction (SPE) [32]. SPE is considered to be one of the most powerful techniques because it minimizes solvent usage and exposure, disposal costs, and extraction time for sample preparation. Several adsorbents have appeared because of the popularity of SPE for selective extraction of analytes, such as alumina [33], C18 [34], cellulose [35], silica gel [36], [37], activated carbon [38], [39], and carbon nanotubes [40], [41].

The current study was aimed to investigate the analytical competition of newly synthesized codoped Ag_2_O_3_-ZnO NCs phase as an adsorbent on the selectivity and adsorption capacity of Co(II) prior to its determination by ICP-OES. The selectivity of NCs Phase towards different metal ions, including Cd(II), Co(II), Cr(III), Cu(II), Fe(III), Ni(II) and Zn(II), was investigated in order to study the effectiveness of doped Ag_2_O_3_-ZnO NCs on the adsorption of selected metal ions. Based on the selectivity study, the NCs are attained the highest selectivity towards Co(II). Static uptake capacity of doped NCs for Co(II) was found to be ∼76.69 mgg^−1^. Adsorption isotherm data of Co(II) with Ag_2_O_3_-ZnO NCs were well-fit with the Langmuir adsorption isotherm, strongly confirmed that the adsorption process was mainly monolayer on homogeneous adsorbent surfaces.

## Experimental Materials and Methods

Zinc chloride (ZnCl_2_), silver chloride (AgCl), sodium hydroxide (NaOH), and all other chemicals were in analytical grade and purchased from Sigma-Aldrich Company. All reagents were used of high purity and spectral purity grades. Doubly distilled de-ionized water was used throughout the measurement such as preparation of the samples and their application. Stock standard solutions of 1000.0 mgL^−1^ Cd(II), Co(II), Cr(III), Cu(II), Fe(III), Ni(II), and Zn(II) were purchased from Sigma-Aldrich (Milwaukee, WI, USA). All reagents were used of high purity and spectral purity grade. Doubly distilled deionized water was used throughout the experimental investigation. The doped Ag_2_O_3_-ZnO NC was investigated with UV/visible spectroscopy (Lamda-950, Perkin Elmer, Germany). FT-IR spectra were recorded for doped Ag_2_O_3_-ZnO NCs with a spectrophotometer (Spectrum-100 FT-IR) in the mid-IR range, which was obtained from Perkin Elmer, Germany. XRD (X'Pert Explorer, PANalytical diffractometer) was equipped with Cu-Kα_1_ radiation (*λ* = 1.5406 nm) by a generator voltage (∼40.0 kV) and current (∼35.0 mA) applied for this measurement. Morphology of Ag-ZnO NCs was checked using FESEM instrument (FESEM; JSM-7600F, Japan). Energy dispersive X-ray analysis (XEDS) of Ag_2_O_3_-ZnO NCs was examined using FESEM-coupled XEDS from JEOL, Japan. The X-ray photoelectron spectroscopy (XPS) measurements were executed for Ag_2_O_3_-ZnO NCs on a Thermo Scientific K-Alpha KA1066 spectrometer (Germany). ICP-OES measurements were acquired by using a Perkin Elmer ICP-OES model Optima 4100 DV, USA. The ICP-OES instrument was optimized daily before measurement and operated as recommended by the manufacturers. The ICP-OES spectrometer was used with following parameters: RF power, 1300 kW; frequency, 27.12 MHz; demountable quartz torch, Ar/Ar/Ar; plasma gas (Ar) flow, 15.0 Lmin^−1^; auxiliary gas (Ar) flow, 0.2 Lmin^−1^; nebulizer gas (Ar) flow, 0.8 Lmin^−1^; nebulizer pressure, 2.4 bar; glass spray chamber according to Scott (Ryton), sample pump flow rate, 1.5 mLmin^−1^; integration time, 3.0 s; replicates, 3; wavelength range of monochromator 165–460 nm. Selected metal ions were measured at wavelengths of 228.80 nm for Cd(II), 238.90 nm for Co(II), 267.72 nm for Cr(III), 327.39 nm for Cu(II), 259.94 nm for Fe(III), 221.65 nm for Ni(II), and 206.20 nm for Zn(II).

### Preparation and growth mechanism of doped Ag_2_O_3_-ZnO NCs

Low-temperature synthesis of Ag_2_O_3_-ZnO NCs was prepared by a wet-chemical process using active reactant precursors such as zinc chloride (ZnCl_2_), silver chloride (AgCl), and sodium hydroxide (NaOH). In a usual reaction procedure, 0.1 M ZnCl_2_ was dissolved in 50.0 ml deionized (DI) water mixed with 50.0 ml AgCl solution (0.1 M) under continuous stirring. pH of resultant solution was adjusted to 10.5 by the addition of NaOH and resulting mixture was shaked and stirred continuously for 30.0 minutes at room conditions. After stirring, the solution mixture was then put into conical flux and heat-up at 150.0°C for 8.0 hours. The temperature of solution was controlled manually throughout the reaction process at 85.0°C. After heating the reactant mixtures, the flux was kept for cooling at room conditions until reached into room temperature. The final Ag_2_O_3_-ZnO doped products were executed, which was washed with DI water, ethanol, and acetone for several times subsequently and dried at room-temperature. The final product was calcined at 400.0°C for three hours in furnace, which was used for structural, elemental, morphological, and optical characterizations. The growth mechanism of the Ag_2_O_3_-ZnO nanocube materials can be explained on the basis of chemical reactions and nucleation, as well as growth of doped nanocrystals. The probable reaction mechanisms are presented here for obtaining the Ag_2_O_3_-ZnO nanomaterials in below. 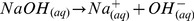
(i)

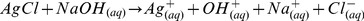
(ii)

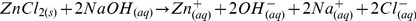
(iii)

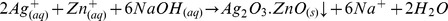
(iv)


**Figure 1:**
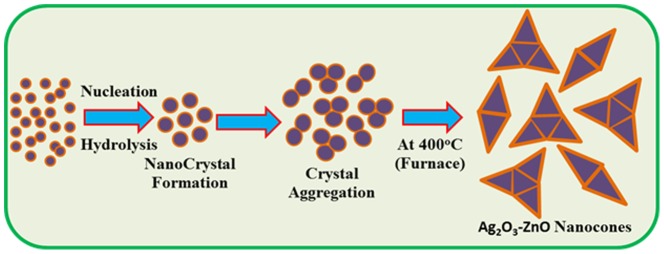
Crystal growth mechanism. Schematic representation of growth mechanism of doped Ag_2_O_3_-ZnO NCs prepared by wet-chemical process.

The reaction is forwarded slowly according to the proposed equation (i) to equation (iii). During preparation, the pH value of the reaction medium plays an important role in the doped nano-material oxide formation. At a particular pH, when AgCl is hydrolyzed with NaOH solution, silver hydroxide is formed instantly according to the equation (ii). During the whole synthesis route, NaOH operates a pH buffer to control the pH value of the solution and slow contribute of hydroxyl ions (OH^-^). When the concentrations of the Ag^+^ and OH^-^ ions are achieved above in critical value, the precipitation of Ag_2_O_3_ nuclei begin to start. As there is high concentration of Zn^2+^ ions [according to the reactions (iii)] in the solution, the nucleation of Ag_2_O_3_ crystals become slower due to the lower activation energy barrier of heterogeneous nucleation. Hence, as the concentration of Zn^2+^ existences, a number of larger Ag_2_O_3_-ZnO crystals with aggregated cube-like morphology form after the reactions [equation (iv)]. The shape of calcined Ag_2_O_3_-ZnO NCs is approximately reliable with the growth pattern of silver oxide codoped zinc oxides nanocrystals [42]–[44]. Then the solution was washed thoroughly with acetone, ethanol, and water successively and kept for drying at room condition. Finally, the as-grown doped Ag_2_O_3_-ZnO NCs materials were calcined at 400.0°C for 8 hours in the furnace (Barnstead Thermolyne, 6000 Furnace, USA). In NCs growth technique, initially Ag_2_O_3_ and ZnO nucleus growth takes place by self-aggregation, which then re-aggregates and produced Ag_2_O_3_-ZnO nanocrystal according to the Ostwald ripening method. Nanomaterial crystallizes and re-aggregates with each other through Vander-Waals forces and forms codoped Ag_2_O_3_-ZnO nanocones morphology, which is presented in Figure 1. Finally, the calcined NCs were fully characterized in detail of their morphological, structural, optical properties, and applied for determination of Co(II) metal ion uptakes for the first time.

### Samples preparation and procedure for metal ions uptake

Stock solutions of Cd(II), Co(II), Cr(III), Cu(II), Fe(III), Ni(II), and Zn(II) were prepared in 18.2 MΩ·cm distilled deionized water and stored in the dark at 4.0°C. For selectivity study of Ag_2_O_3_-ZnO NCs phase toward various metal ions, standard solutions of 5.0 mgL^−1^ metal ion were prepared and individually mixed with 25.0 mg of the Ag_2_O_3_-ZnO NCs phase. By ICP-OES analysis, it has been performed experimental investigation on the liquid eluates, which separated from the solid NCs. For the effect of pH on the selectivity of Ag_2_O_3_-ZnO NCs for Co(II), 5.0 mgL^−1^ Co(II) standard solutions were prepared and adjusted to pH values ranging from 1.0 to 9.0 with appropriate buffer solutions, 0.2 molL^−1^ HCl/KCl for pH 1.0 and 2.0, 0.1 molL^−1^ CH_3_COOH/CH_3_COONa for pH 3.0–6.0 and 0.1 molL^−1^ Na_2_HPO_4_/HCl for pH 7.0–9.0. Each standard solution was individually mixed with 25.0 mg Ag_2_O_3_-ZnO NCs phase. The adsorbent dose was fixed at 25.0 mg because minimum to no change was noticed in the uptake capacity of Ag_2_O_3_-ZnO NCs for Co(II) with adsorbent dose above 25.0 mg under batch conditions. All mixtures were shaken vigorously at room temperature for 1.0 h. The supernatant concentrations of metal ions were directly estimated by ICP-OES after filtration. For investigation of the Co(II) uptake capacity, standard solutions of 0, 5.0, 10.0, 15.0, 20.0, 25.0, 30.0, 50.0, 75.0, 125.0, and 150.0 mgL^−1^ were prepared as above, adjusted to the optimum pH value of 5.0 and individually mixed with 25.0 mg Ag_2_O_3_-ZnO NCs. All mixtures were mechanically shaken for 1.0 hr at room temperature. In addition, the effect of shaking time on Co(II) adsorption on Ag_2_O_3_-ZnO NCs phase was evaluated under the same batch conditions but at different equilibrium periods (2.5, 5, 10, 20, 30, 40, 50 and 60 min). For the effect of temperature on the adsorption of Ag_2_O_3_-ZnO NCs toward Co(II), standard solutions of 5.0 mgL^−1^ Co(II) were prepared, adjusted to pH 5.0 as above and individually mixed with 25.0 mg Ag_2_O_3_-ZnO NCs. The effect of temperature on the adsorption of Ag_2_O_3_-ZnO NCs toward Co(II) was also investigated under the same batch conditions but at different temperatures (278, 298, 313, and 338 K).

## Results and Discussion

### Morphology and structural evaluation

**Figure 2:**
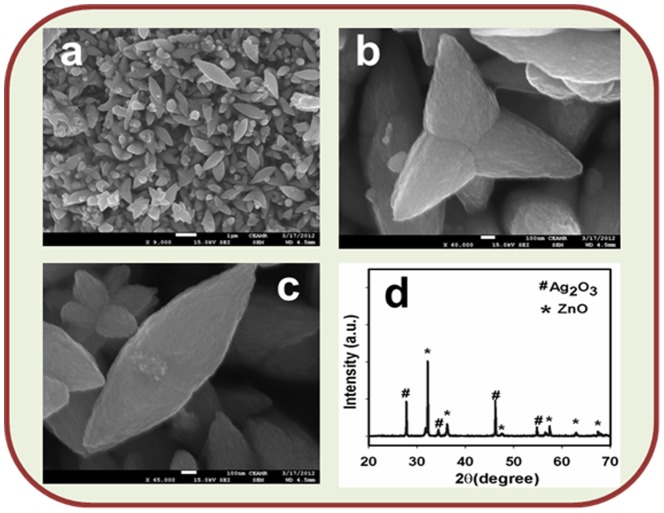
Studies of Morphology and Crystallinity. (a–c) FESEM images and (d) Powder x-ray diffraction pattern of calcined Ag_2_O_3_-ZnO NCs at room conditions.

High resolution FESEM images of calcined Ag_2_O_3_-ZnO NCs are presented in Figure 2(a–c). The FESEM images display of codoped nano-structural materials with aggregated nano-cube shapes. The average length and cross-section (center) of Ag_2_O_3_-ZnO NCs is calculated as 1.42 µm and 0.53 µm respectively. It is exhibited perceptibly from the FESEM images that the simple wet-chemically technique of prepared doped products are nanocubes of Ag_2_O_3_-ZnO. It is revealed in aggregated arrangement with high-density and obtained nanostructure in round cubic-shapes. It is also proposed that approximately all of the nanostructure composed in cone-like shapes of the aggregated Ag_2_O_3_-ZnO NCs [45]. Crystallinity and crystal phase of calcined Ag_2_O_3_-ZnO NCs were investigated. Powder X-ray diffraction patterns of doped nanocubes are represented in Figure 2d. The Ag_2_O_3_-ZnO NCs sample were investigated and exhibited as face-centered cubic shapes. Figure 1d reveals characteristic crystallinity of the Ag_2_O_3_-ZnO NCs and their crystalline arrangement, which is investigated by powder X-ray crystallography. Separately, all the reflection peaks in this prototype were related with ZnO phase having face-centered cubic zincite geometry [Joint Committee on Powder Diffraction Standards; JCPDS # 01-074-9942]. The phases demonstrated the key features with indices for crystalline ZnO at 2θ values of 32.1 (100), 36.7 (101), 47.9 (102), 58.1 (110), 62.8 (103), and 68.2 (200) degrees. The face-centered cubic lattice parameters are a = 3.2049, c = 5.2038, Z = 2, and radiation (CuK_α_1, λ = 1.5406). The ZnO phases have a high degree of crystallinity. All of the peaks match well with Bragg reflections of the standard zincite structure (point or space-group P63mc) [46]. The reflection peaks were also found to correspond with Ag_2_O_3_ phase having face-centered cubic orthorhombic geometry [JCPDS #01- 077-1829]. The phases demonstrated the key features with indices for crystalline Ag_2_O_3_ at 2θ values of 27.9 (120), 33.6 (031), 46.3 (032), and 55.1 (800) degrees. The Ag_2_O_3_ phases have a high degree of crystallinity. All of the peaks match well with Bragg reflections of the standard orthorhombic structure. The lattice parameters of Ag_2_O_3_ are a = 12.869, b = 10.49, c = 3.6638. These confirmed that there is major number and amount of crystalline doped Ag_2_O_3_-ZnO present in NCs [47], [48].

Crystallite size was calculated by Debye-Scherrer's formula given by equation (v)
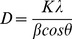
(v)


Where D is the crystal size; λ is the wavelength of the X-ray radiation (λ = 0.15406 nm) for CuKα; K is usually taken as 0.9; and β is the line width at half-maximum height (FWHM) [49]. The average cross sectional diameter of Ag_2_O_3_-ZnO NCs is close to ∼0.51 µm.

### Optical and elemental evaluation

**Figure 3:**
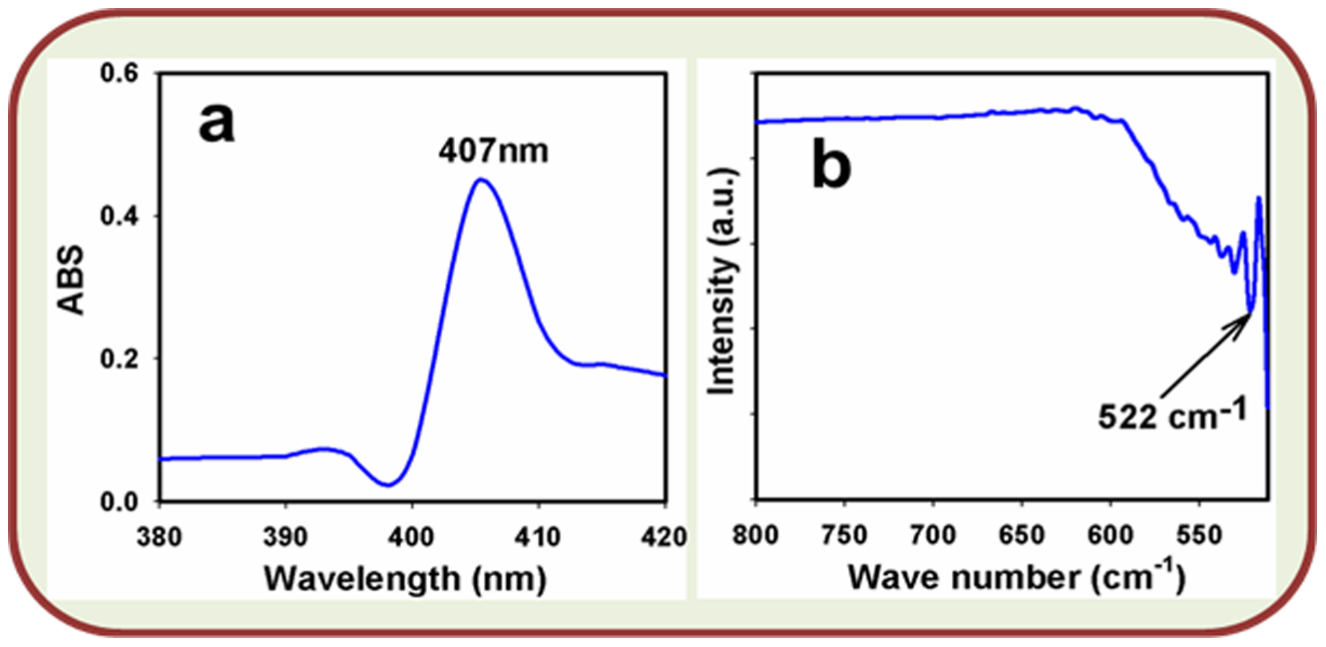
Studies of Optical property. (a) UV/visible spectroscopy and (b) FT-IR spectroscopy of calcined Ag_2_O_3_-ZnO NCs.



The optical property of calcined Ag_2_O_3_-ZnO NCs is one of the important characteristics for the evaluation of its photo-catalytic activity. The optical absorption spectra of Ag_2_O_3_-ZnO NCs are accomplished by using UV-vis. spectrophotometer in the visible range (200.0∼800.0 nm). UV/visible system are a method in which the outer electrons of atoms or molecules absorb radiant energy and undergo transitions to high energy levels. In this method, the spectrum is obtained due to optical absorption can be analyzed to obtain the energy band-gap (E_bg_) of the semiconductor doped nanomaterials. The optical absorption measurement was carried out at ambient conditions. From the absorption spectrum, it has been found the maximum wavelength for the calcined Ag_2_O_3_-ZnO NCs is about 407.0 nm, which is presented in Figure 3a. Bang-gap energy (E_bg_) is calculated on the basis of the maximum absorption band of NCs and found to be ∼3.0467 eV, according to following equation (vi).

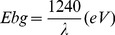
(vi)Where E_bg_ is the band-gap energy and λ_max_ is the wavelength (∼407.0 nm) of the NCs. No extra peak associated with impurities and structural defects are observed in the spectrums, which proved that the synthesized NCs control crystallinity of Ag_2_O_3_-ZnO materials [50], [51].

The calcined Ag_2_O_3_-ZnO NCs are also measured in term of the atomic and molecular vibrations. To predict the functional-group recognition, FT-IR spectra are investigated in the region of 400∼4000 cm^−1^at room conditions. Figure 3b displays the FT-IR spectrum of the NCs. It represents band at 522 cm^−1^. The observed broad vibration band (at 522 cm^−1^) could be assigned as metal-oxygen (Ag-O & Zn-O mode) stretching vibrations, which demonstrated the configuration of Ag_2_O_3_-ZnO NCs materials. [52], [53]. Hence, the experimental vibration bands at low frequencies regions (at 522.0 cm^−1^) recommended the formation of Ag_2_O_3_-ZnO NCs by a wet-chemical method.

**Figure 4:**
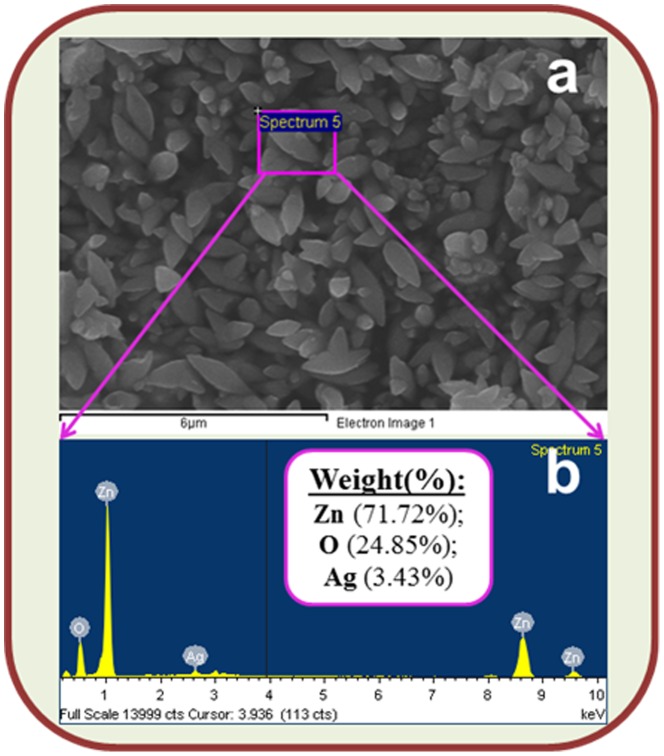
Studies of Elemental Analysis. (a) Ag_2_O_3_-ZnO NCs image of FESEM coupled XEDS and (b) elemental spectrum of XEDS. (Inset: elemental composition weight percent).

The X-ray electron dispersive spectroscopy (XEDS) analysis of these Ag_2_O_3_-ZnO NCs are indicated the presence of silver (Ag), zinc (Zn), and oxygen (O) composition in the pure calcined nanostructure material, which is presented in Figure 4a. It is clearly displayed that the calcined prepared nanomaterials contained only Ag, Zn, and O elements with the 3.43, 71.72, 24.85 wt% respectively, which is presented in Figure 4b and inset of Figure 4b. No other peak related with any impurity has been detected in the FESEM coupled XEDS, which confirms that the nanocones are composed only with Ag, Zn, and O.

**Figure 5:**
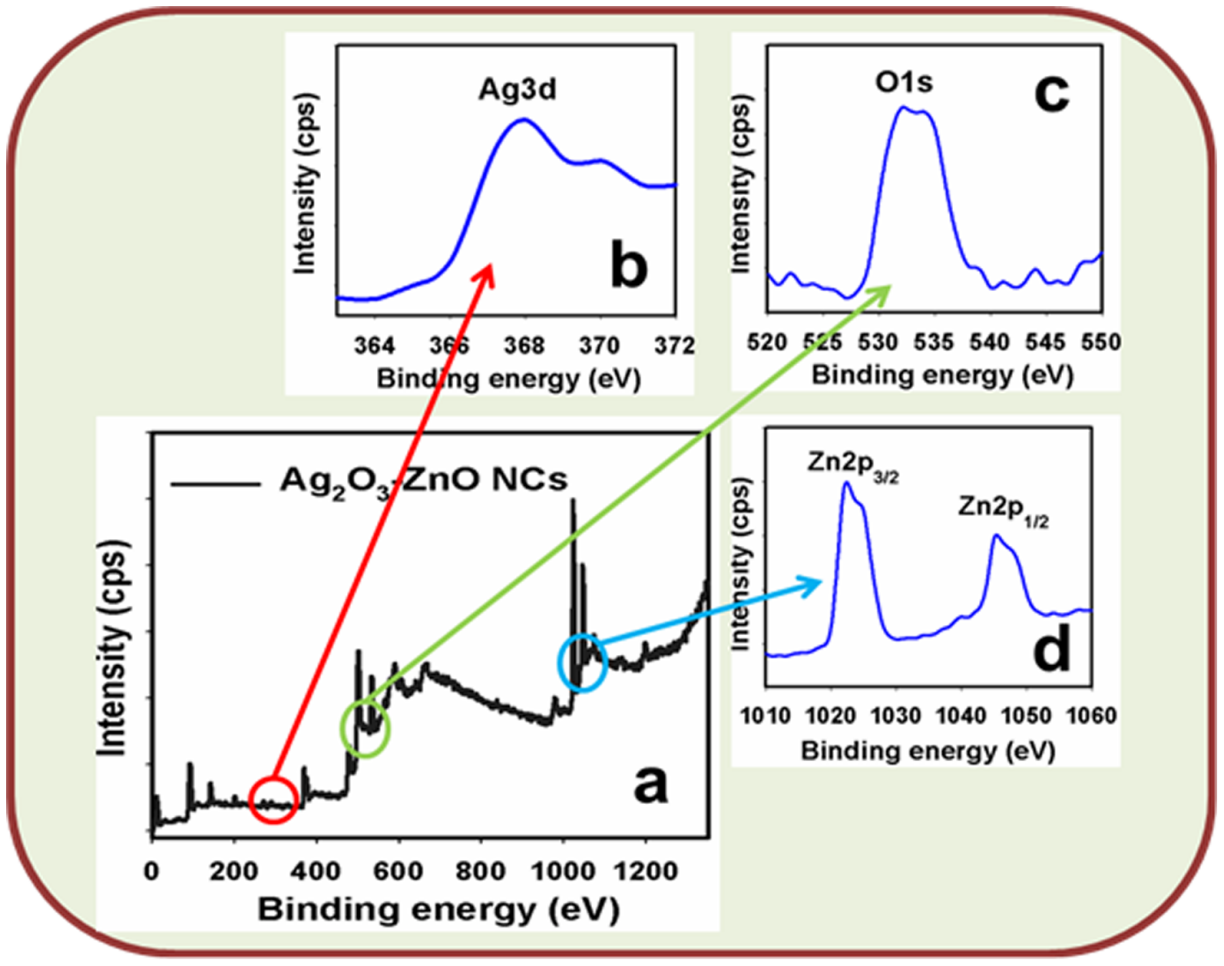
Studied of Binding energy. XPS of (a) doped Ag_2_O_3_-ZnO NCs, (b) Ag3d level, (c) O1s level, and (d) Zn2p level acquired with MgKα radiation.

X-ray photoelectron spectroscopy (XPS) is a quantitative spectroscopic technique that determines the chemical-states of the elements that present within doped materials. XPS spectra are acquired by irradiating on an Ag_2_O_3_-ZnO NCs with a beam of X-rays, while simultaneously determining the kinetic energy and number of electrons that get-away from the top one to ten nm of the material being analyzed. Here, XPS measurements were measured for Ag_2_O_3_-ZnO NCs semiconductor nanomaterials to investigate the chemical states of ZnO and Ag_2_O_3_. The XPS spectra of Ag3d, Zn2p, and O1s are presented in Fig. 5a. XPS was also used to resolve the chemical state of the doped Ag_2_O_3_ nanomaterial and their depth. Figure 5b presents the XPS spectra (spin-orbit doublet peaks) of the Ag3d_(5/2)_ regions recorded with semiconductor doped materials. The binding energy of the Ag3d_(5/2)_ peak at 368.2 eV denotes the presence of Ag_2_O_3_ since their bindings energies are similar [54]. The O1s spectrum shows a main peak at 532.1 eV in Fig. 5c. The peak at 532.1 eV is assigned to lattice oxygen may be indicated to oxygen (ie, O_2_^-^) presence in the doped Ag_2_O_3_-ZnO NCs [55]. In Figure 5d, the spin orbit peaks of the Zn2p_(1/2)_ and Zn2p_(3/2)_ binding energy for all the samples appeared at around 1024.5 eV and 1048.1 eV respectively, which is in good agreement with the reference data for ZnO nanomaterials [56]. XPS compositional analyses evidenced the co-existence of the two single-phase of Ag_2_O_3_ and ZnO nanomaterials. Therefore, it is concluded that the wet-chemically prepared doped Ag_2_O_3_-ZnO materials have NCs phase contained two materials. Also, this conclusion is reliable with the XRD data noticeably.

### Detection of cobalt ions using batch method

#### Selectivity study of Ag_2_O_3_-ZnO NCs

Selectivity of the newly synthesized phase toward different metal ions was investigated based on the basis of calculated distribution coefficient of doped Ag_2_O_3_-ZnO NCs phase. The distribution co-efficient (*K_d_*) can be obtained from the following equation (vii) [57]: 
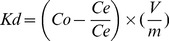
(vii)where *C_o_* and *C_e_* refer to the initial and final concentrations before and after filtration with Ag_2_O_3_-ZnO NCs, respectively, *V* is the volume (mL) and *m* is the weight of Ag_2_O_3_-ZnO NCs phase (g). Distribution coefficient values of all metal ions investigated in this study are summarized in **Table 1**. It can be clearly observed from **Table 1** that the greatest distribution coefficient value was obtained for Co(II) with Ag_2_O_3_-ZnO NCs in comparison to other metal ions. As can be depicted from Table1, the amount of Co(II) was almost all extracted by Ag_2_O_3_-ZnO NCs phase. Thus, selectivity study results indicated that the newly synthesized Ag_2_O_3_-ZnO NCs phase was most selective toward Co(II) among all metal ions.

**Table 1:** Selectivity study of the adsorption of Ag_2_O_3_-ZnO NCs phase toward different metal ions at pH 5.0 and 25°C (*N* = 3).

Metal Ions	*q_e_* (mgg^−1^)	*K_d_* (mLg^−1^)
**Co(II)**	4.90	49505.05
**Ni(II)**	3.31	1960.33
**Cd(II)**	0.61	138.95
**Cr(III)**	0.60	137.40
**Fe(III)**	0.29	61.35
**Zn(II)**	0.02	4.02
**Cu(II)**	0.01	2.00

### Effect of pH on Ag_2_O_3_-ZnO NCs uptake for Co(II)

**Figure 6:**
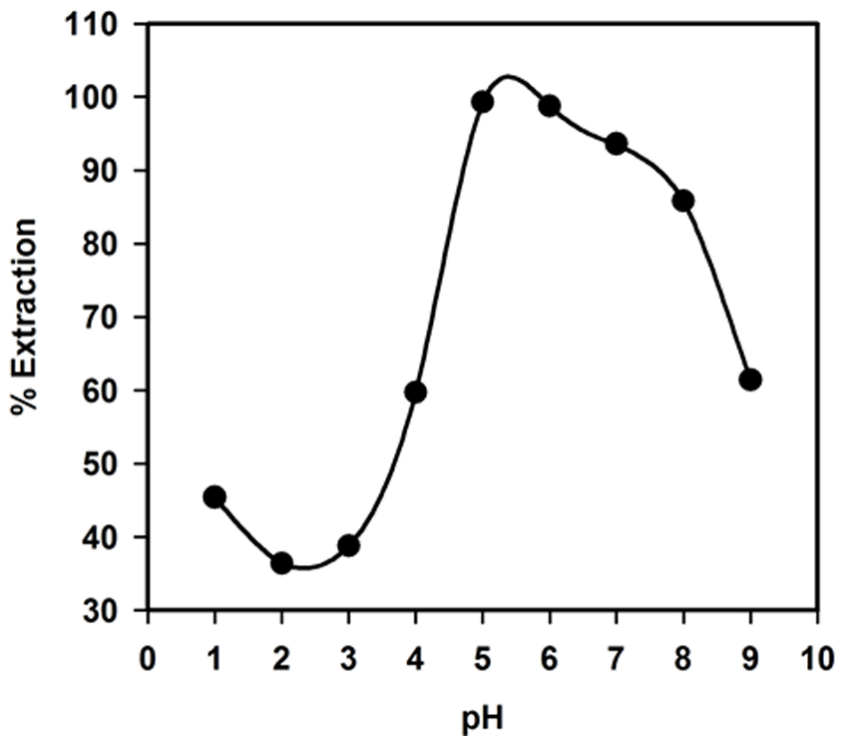
pH effect. Effect of pH on the adsorption of 5.0 mgL^−1^ Co(II) on 25.0 mg Ag_2_O_3_-ZnO NCs phase at 25.0°C.

The effect of pH on the adsorption of Ag_2_O_3_-ZnO NCs phase toward 5.0 mgL^−1^ Co(II) was studied in the range of 1.0–9.0 (Figure 6). As illustrated in Figure 6, there is an increase followed by a subsequent decrease in % extraction of Co(II) with an increase in the pH. A close examination of Figure 6 indicates that almost all Co(II) was extracted at pH 5.0, providing that the adsorption process may be electrostatic attraction or complex formation. Based on the above results, pH 5.0 was selected to be the optimum pH value for studying other parameters influencing the uptake capacity of Ag_2_O_3_-ZnO NCs for Co(II) under batch conditions.

**Figure 7:**
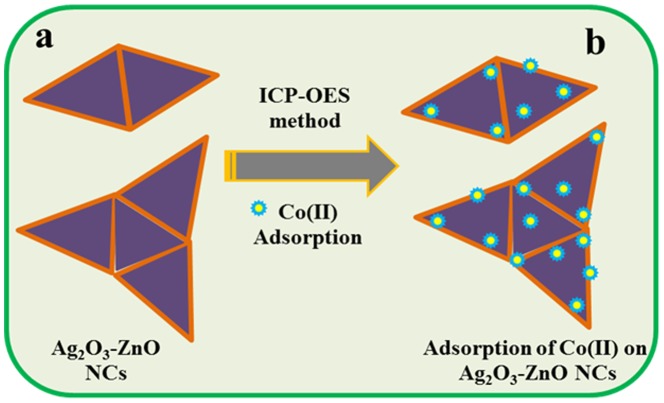
Studied of selective ionic Adsorption. Schematic representation of before (a) and after (b) selective Co(II) adsorption onto doped Ag_2_O_3_-ZnO NCs.

A schematic diagram of Co(II) ion adsorption on the Ag_2_O_3_-ZnO NCs is presented in the Figure 7. In the scheme, it is shown the adsorption of Co(II) ions (before and after) onto doped Ag_2_O_3_-ZnO NCs [Fig. 7a and Fig. 7b]. From these results, it can be noticed that the selectivity of prepared doped Ag_2_O_3_-ZnO NCs phase toward Co(II) was the most among all metal ions. Thus, Ag_2_O_3_-ZnO NCs phase can be selectively bound by Co(II) ions, providing that the mechanism of adsorption may be electrostatic attraction or complex formation.

### Static adsorption capacity

**Figure 8:**
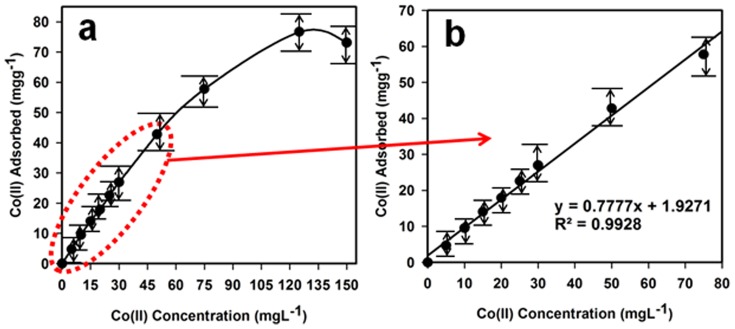
Studied of Adsorption behavior. (a) Adsorption profile and (b) calibration plot of Co(II) on 25.0 mg Ag_2_O_3_-ZnO NCs phase in relation to the concentration at pH 5.0 and 25.0°C.

For determination of the static uptake capacity of Co(II) on Ag_2_O_3_-ZnO NCs adsorbent 25.0 mL Co(II) sample solutions with different concentrations (0∼150.0 mgL^−1^) were adjusted to pH 5.0 and individually mixed with 25.0 mg Ag_2_O_3_-ZnO NCs. These mixtures were mechanically shaken for 1 hr at room temperature. Static adsorption capacity was obtained using equation (viii) as follows:
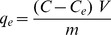
(viii)where *q*_e_ represents the adsorbed metal ion by the adsorbent (mgg^−1^), C_ο_ and C_e_ are the initial and equilibrium concentrations of metal ion in solution (mgL^−1^), respectively, *V* is the volume (L) and *m* is the weight of adsorbent (g). Figure 8a displays the metal uptake capacity of Ag_2_O_3_-ZnO NCs phase for Co(II) obtained from the experiment of adsorption isotherm. Adsorption capacity of Ag_2_O_3_-ZnO NCs for Co(II) was determined to be 76.69 mgg^−1^. Reported adsorption capacity in this study was found to be comparable with those previously reported for Co(II) ion (0.92–1.69) [58], 2.90 [59], 11.53 [60], 19.75 [61], 24.75 [62] and other studies [63]–[65]. The sensitivity (slope) and linearity (R^2^) of Co(II) using Ag_2_O_3_-ZnO NCs phase is calculated from the calibration plot (Figure 8b), which is close to 0.7777 L.g^−1^ and 0.9928 respectively.

### Adsorption isotherm models

Experimental equilibrium adsorption data were analyzed using different models in order to develop an equation that accurately represents the results. Langmuir equation was based on an assumption of a monolayer adsorption onto a completely homogeneous surface with a finite number of identical sites and a negligible interaction between the adsorbed molecules. The Langmuir adsorption isotherm model was governed by the following relation (ix) [66]:
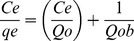
(ix)where *C*_e_ corresponds to the equilibrium concentrations of Co(II) ion in solution (mgmL^−1^) and *q*_e_ is the adsorbed metal ion by the adsorbate (mgg^−1^). The symbols *Q*_o_ and *b* refer to Langmuir constants related to adsorption capacity (mgg^−1^) and energy of adsorption (Lmg^−1^), respectively. These constants can be determined from a linear plot of *C*_e_/*q*_e_ against *C*_e_ with a slope and intercept equal to 1/*Q*_o_ and 1/*Q*_o_ *b*, respectively. Moreover, the essential characteristics of Langmuir adsorption isotherm can be represented in terms of a dimensionless constant separation factor or equilibrium parameter, *R_L_*, which is defined as *R_L_* = 1/(1+*bC*_o_), where *b* is the Langmuir constant (indicates the nature of adsorption and the shape of the isotherm); *C*_o_ the initial concentration of the analyte. The *R_L_* value indicates the type of the isotherm, and *R_L_* values between 0 and 1 represent a favorable adsorption [67].

**Figure 9:**
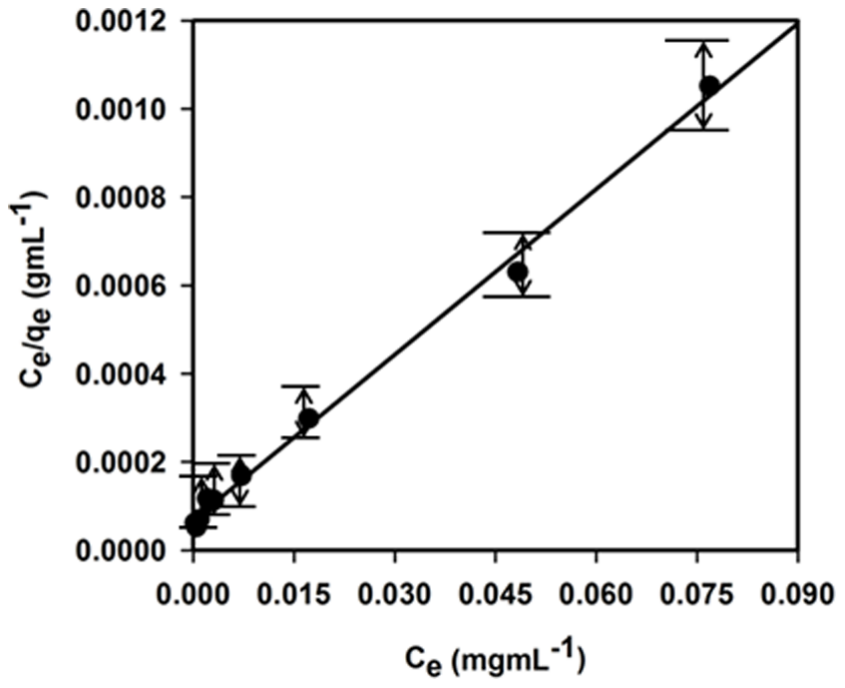
Studied of Langmuir-Adsorption. Langmuir adsorption isotherm model of Co(II) adsorption on 25 mg Ag_2_O_3_-ZnO NCs adsorbentat pH 5.0 and 25°C. Adsorption experiments were obtained at different concentrations (0–150.0 mgL^−1^) of under static conditions.

The experimental isotherm data were best fit with the Langmuir equation (Figure 9) based on the least square fit, confirming the validity of Langmuir adsorption isotherm model for the adsorption process. Consequently, adsorption isotherm data suggested that the adsorption process was mainly monolayer on a homogeneous adsorbent surface. Langmuir constants *Q*_o_ and *b* are found to be 79.93 mgg^−1^ and 0.18 Lmg^−1^, respectively. The correlation coefficient obtained from the Langmuir model is found to be *R^2^* = 0.9960 for adsorption of Co(II) on doped Ag-ZnO NCs. Moreover, the Co(II) adsorption capacity (79.93 mgg^−1^) calculated from Langmuir equation was consistent with that (76.69 mgg^−1^) of the experimental isotherm study. The *R_L_* value of Co(II) adsorption on the Ag_2_O_3_-ZnO NCs is 0.04, supporting a highly favorable adsorption process based on the Langmuir classical adsorption isotherm model.

### Effect of shaking time

**Figure 10:**
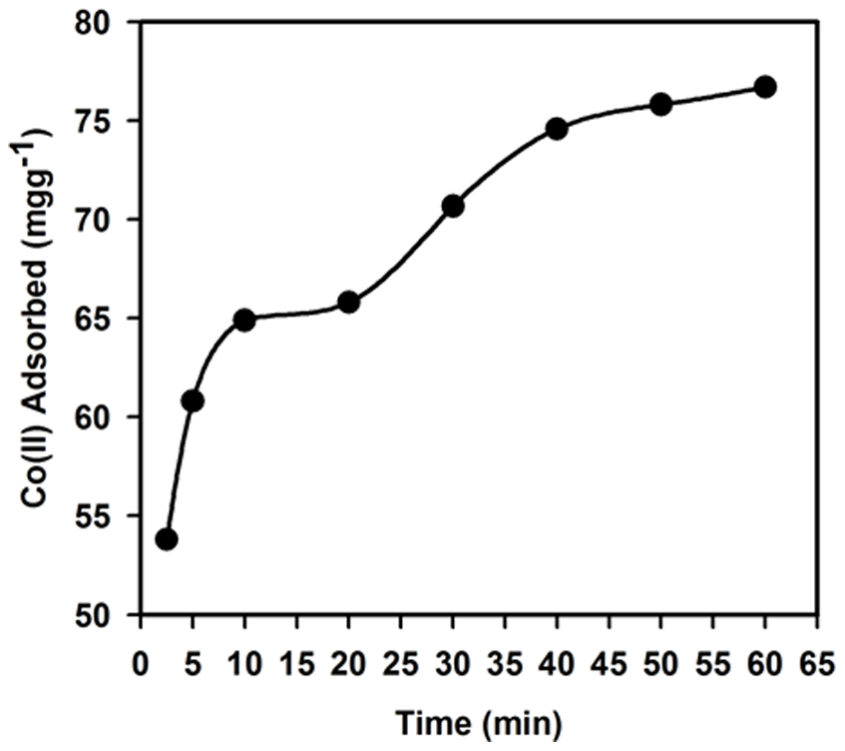
Studies of Adsorption characteristic. Effect of contact time on the adsorption of 125.0 mgL^−1^ Co(II) on 25.0 mg Ag_2_O_3_-ZnO NCs at pH 5.0 and 25.0°C.

In order to assess the possibility of applications for the Ag_2_O_3_-ZnO NCs to selectively bind Co(II) and estimate the time required to attain equilibrium, the effect of contact time was evaluated. The batch procedure was implemented at different contact times, ranging from 2.5 to 60.0 min, and at a fixed concentration of 125.0 mgL^−1^ Co(II), as shown in Figure 10. Figure 10 indicates that the amount of Co(II) adsorbed on Ag_2_O_3_-ZnO NCs phase was dramatically increased with an increase of the contact time. More than 64.0 mgg^−1^ Co(II) was adsorbed on Ag_2_O_3_-ZnO NCs after only 10.0 min of the equilibrium period. The uptake capacity of Co(II) was also increased to more than 70.0 mgg^−1^ after 30 min until the maximum adsorption of Ag_2_O_3_-ZnO NCs for Co(II) was reached after 60 min. Thus, it was clear that equilibrium kinetics for Co(II) adsorption on Ag_2_O_3_-ZnO NCs phase was very fast.

### Kinetic models

Different kinetic models were investigated in order to explore inherent kinetic adsorption parameters of the sorbate-adsorbent system. Kinetic models were used to evaluate fitness of experimental data in which correlation coefficient (*R^2^*) value provides the measure of agreement between the experimental data. The adsorption kinetic equation of a pseudo second-order adsorption can be given as follows (x):
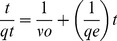
(x)where *υ*_o_ =  *k_2_* 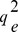 is the initial adsorption rate (mgg^−1^min^−1^) and *k_2_* (gmg^−1^min^−1^) denotes the rate constant of adsorption, *q*_e_ (mgg^−1^) corresponds to the amount of metal ion adsorbed at equilibrium, and *q*_t_ (mgg^−1^) represents the amount of meal ion on the adsorbent surface at any time *t* (min). The parameters *q*_e_ and *υ*_o_ can be estimated from the slope and intercept, respectively, of a plot of *t*/*q*_t_ versus *t* [68]–[72].

**Figure 11:**
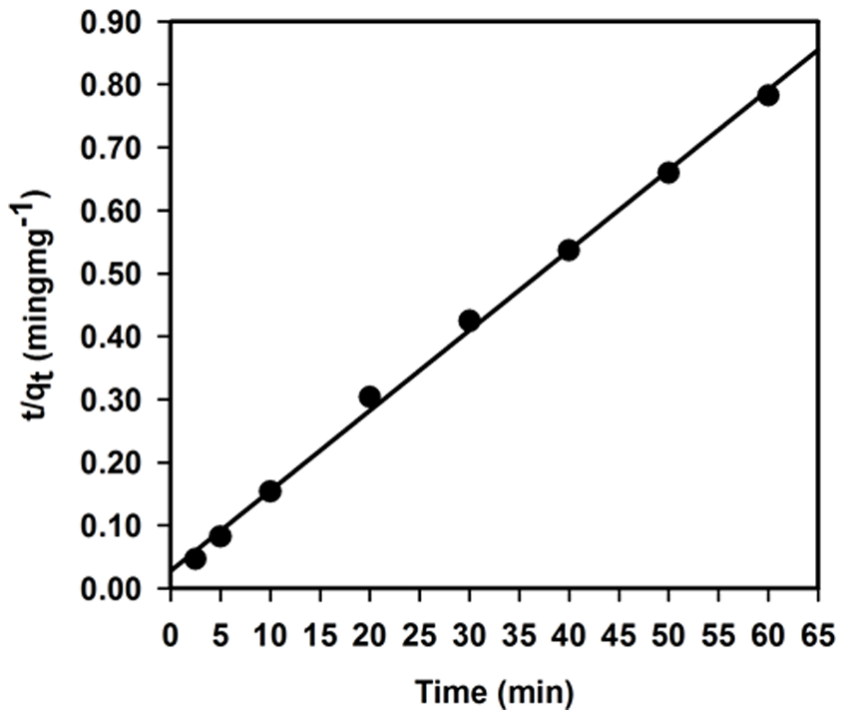
Studies of adsorption kinetics. Pseudo second-order adsorption kinetic model of Co(II) uptake on 25.0 mg Ag_2_O_3_-ZnO NCs at pH 5.0 and 25.0°C.

Adsorption kinetics data were well fit with the pseudo second-order model, providing that the kinetics of Co(II) adsorption on Ag_2_O_3_-ZnO NCs followed the pseudo second-order kinetic (Figure 11). The value of *R^2^* (0.9980) also confirmed that the pseudo second-order model was more reliable and accurate as compared to other kinetic models. Parameters *υ*_o_, *q*_e_ and *k_2_* were estimated to be 36.39 mgg^−1^min^−1^, 78.53 mgg^−1^ and 0.01 gmg^−1^min^−1^, respectively. It can be clearly observed that the observed value of *q*_e_ obtained from the pseudo second-order kinetics model was in agreement with that of adsorption isotherm experiments, strongly confirming the validity of pseudo second-order kinetics model.

### Effect of temperature

The effect of temperature on the adsorption of Ag_2_O_3_-ZnO NCs phase for Co(II) was evaluated in order to determine thermodynamic parameters. Thermodynamic parameters of the adsorption of 25.0 mg Ag_2_O_3_-ZnO NCs toward 5.0 mgL^−1^ Co(II) was studied at different temperatures (278, 298, 313, and 338 K). The distribution adsorption coefficient (*K_d_*) corresponding to the character of a metal ion adsorbed by an adsorbent (mLg^−1^) can be obtained from equation (xi). Thermodynamic parameters of the standard enthalpy change (Δ*H°*, kJmol^−1^) and standard entropy change (Δ*S°*, Jmol^−1^K^−1^) were estimated, as recorded in **Table 2**, from the slope and intercept, respectively, of the linear variation of *lnK_d_* with the reciprocal of temperature (*1/T*) as follows:
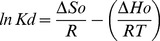
(xi)


**Table 2:** Calculated thermodynamic parameters of 5.0 mgL^−1^ Co(II) adsorption on 25.0 mg Ag_2_O_3_-ZnO NCs (*N* = 3).

Δ*H°* (kJmol^−1^)	Δ*S°* (Jmol^−1^ K^−1^)	Δ*G°* (kJmol^−1^)			
		*T* = 278 K	*T* = 298 K	*T* = 313 K	*T* = 338 K
–23.14	19.93	−28.61	−29.25	−29.33	−29.85

where *R* refers to the universal gas constant (∼8.314 Jmol^−1^K^−1^), and *T* denotes the temperature in Kelvin. The standard Gibbs free energy change (Δ*G°*, kJmol^−1^) reported in **Table 2** was calculated from the following equation (xii):
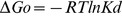
(xii)


The estimated values of standard enthalpy change, Δ*H°*, and Gibbs free energy change, Δ*G°*, are negative, while that of the standard entropy change, Δ*S°*, is positive. The observed negative Δ*H°* value suggested an exothermic adsorption of Co(II) on Ag_2_O_3_-ZnO NCs phase. The negative Δ*G°* together with positive Δ*S°* suggested that the adsorption mechanism of Ag_2_O_3_-ZnO NCs for Co(II) is a general spontaneous process and thermodynamically favorable because. The positive value of Δ*S°* also indicates that the degree of freedom increases at the solid–liquid interface during the adsorption of Co(II) on Ag_2_O_3_-ZnO NCs. These results strongly supported those concluded from adsorption isotherm experiments, Langmuir and kinetic adsorption isotherm models.

### Performance of method in analytical applications

#### Effect of salt addition on the adsorption and extraction of Co(II)

For an assessment of the possibility of analytical applications for the proposed procedure in analyzing real samples, the effect of different ions was evaluated under optimized conditions. Model standard solutions containing fixed amount of 1.0 mgL^−1^ Co(II) together with either individual or mixed matrix ions were prepared according to the recommended procedure. Results summarized in **Table 3** indicated that the extraction of Co(II) was not affected by the medium composition containing either individual or mixed ions. This may be attributed to the low uptake capacity or rate for interfering ions toward Ag_2_O_3_-ZnO NCs phase. Thus, it can be concluded that the Ag_2_O_3_-ZnO NCs has high selectivity toward Co(II) when compared to other interfering ions, and the proposed method can be applied for determination of Co(II) in real environmental samples.

**Table 3:** Effect of matrix interferences on the extraction of 1.0 mgL^−1^ Co(II) on 25.0 mg Ag_2_O_3_-ZnO NCs (*N* = 3).

Coexisting ions	Concentration (mgL^−1^)	% Extraction of Co(II)
**Na^+^, K^+^, NH_4_^+^**	3000	98.07
**Ca^2+^, Mg^2+^, Ba^2+^**	1000	97.44
**Cd^2+^**	400	93.33
**Cu^2+^**	400	96.32
**Pb^2+^**	500	94.11
**Mn^2+^**	300	97.21
**Fe^3+^**	500	92.05
**Al^3+^**	300	91.55
**Cl^−^, F^−^, NO_3_^−^**	2000	98.45
**CO_3_^2−^, SO_4_^2-^**	1500	96.64
**PO_4_^3−^**	1000	95.91

### Application of the proposed method (real-samples analysis)

The proposed method was implemented to the determination of Co(II) in real water samples. A standard addition method was used to evaluate the accuracy of the Co(II) extraction in four types of water samples, including drinking water, lake water, seawater, and tap water, collected from Jeddah in Saudi Arabia. The percent (%) extraction of different amounts of Co(II) in real water samples was obtained, as presented in **Table 4**. Results showed that the % extraction of Co(II) in spiked water samples was in the range of 91.55–98.45%. Thus, the proposed method was apparently reasonable and reliable for trace analysis is for analyzing real samples.

**Table 4:** Determination of Co(II) at different concentrations in real water samples using 25.0 mg Ag_2_O_3_-ZnO NCs (*N* =  3).

Samples	Added (mgL^−1^)	Un-adsorbed (mgL^−1^)	Extraction (%)
	2	0.05	97.40
**Tap water**	6	0.24	96.02
	12	0.65	94.58
	2	0.07	96.26
**Lake water**	6	0.27	95.52
	12	0.86	92.80
	2	0.08	95.87
**Sea water**	6	0.36	93.92
	12	1.20	90.03
**Drinking Water**	2	0.02	98.85
	6	0.14	97.75
	12	0.56	95.34

## Conclusions

The doped Ag_2_O_3_-ZnO NCs are successfully prepared by a wet-chemically coupled with a heat-treatment method at low-temperature and characterized in detail in terms of their elemental, morphological, structural, and optical properties. The doped Ag_2_O_3_-ZnO NCs nanostructures are possessed in hexagonal geometry. The proposed method confirmed the efficiency of the newly prepared Ag_2_O_3_-ZnO NCs phase for a selective adsorption and determination of Co(II) in aqueous media at short contact time. Reasonable static adsorption capacities of ∼76.69 mgg^−1^ for Ag_2_O_3_-ZnO NCs adsorbent was achieved for Co(II) in aqueous solution. Adsorption isotherm data of Co(II) were well fit with the Langmuir classical adsorption isotherm model. Results also demonstrated that the adsorption of Ag_2_O_3_-ZnO NCs toward Co(II) obeyed a pseudo second-order kinetic model. Based on thermodynamic study, the adsorption process of Co(II) on Ag_2_O_3_-ZnO NCs was a general spontaneous process and thermodynamically favorable. Moreover, the extraction of Co(II) by Ag_2_O_3_-ZnO NCs was not affected by the medium composition containing either individual or mixed ions. Thus, the proposed method was found to be reliable, feasible and applicable to real environmental samples analysis.
